# Proximal Interruption of Pulmonary Artery: Spectrum of Radiological Findings With Emphasis on Chest Radiograph and Contrast-Enhanced Computed Tomography (CECT)

**DOI:** 10.7759/cureus.32916

**Published:** 2022-12-25

**Authors:** Raja Kollu, Seema Uligada, Sai Kotamraju, Bhavana Lakshmi Nethi Balingari, Anees Dudekula, Prashanth Kumar, Chandrashekar Patil, Shubham Gaddalay, Saumya L Gaddalay

**Affiliations:** 1 Radiodiagnosis, Malla Reddy Medical College for Women, Hyderabad, IND; 2 Radiodiagnosis, Vydehi Institute of Medical Sciences and Research Centre, Bengaluru, IND; 3 Radiodiagnosis, East Point Medical College, Bengaluru, IND; 4 Internal Medicine, Gandhi Medical College, Hyderabad, IND; 5 Internal Medicine, Malla Reddy Medical College for Women, Hyderabad, IND

**Keywords:** extrapulmonary arteries from the aorta, truncus arteriosus, patent ductus arteriosus, tetralogy of fallot, scimitar sign, mapcas, major aortopulmonary collateral arteries, proximal interruption of pulmonary artery

## Abstract

Introduction

Proximal interruption of pulmonary artery (PIPA) is a congenital anomaly presenting with aberrant termination of the pulmonary artery at the hilum. It results in a variety of radiological and clinical manifestations. Clinically, isolated PIPA can be asymptomatic till late adulthood or can present with dyspnoea, chest discomfort, hemoptysis and recurrent infections. PIPA can be associated with multiple cardiovascular anomalies such as tetralogy of Fallot (TOF), ventricular septal defects (VSD), and scimitar syndrome. We present a spectrum of cases with both isolated proximal interruption of the pulmonary artery and cases associated with other cardiovascular abnormalities. Typical chest radiographs and chest contrast-enhanced computed tomography (CECT) findings are discussed and demonstrated in detail. Proper and early diagnosis is a crucial step as it can significantly modify the treatment choice, thereby reducing morbidity.

Objective

To document the different presentations of the proximal arrest of pulmonary arteries, to document associations with cardiovascular and pulmonary manifestations, and to elaborate on and demonstrate the various radiological imaging findings.

Material and methods

All the cases that were reported with proximal interruption of pulmonary artery on the CECT studies conducted between 2019 and 2022 at a tertiary care hospital in Telangana, India. The demographic data, clinical presentation, chest radiographs, and chest CECT were collected retrospectively. Data analysis was done using Microsoft Excel 2019 to calculate descriptive statistics. A total of 22 cases were identified of which three cases were excluded of as they were previously operated and 19 cases were taken as the study population.

Results

Nineteen patients were included in the study. Demographic details, clinical history, CECT, and chest radiographs were collected wherever available. The majority of the cases belonged to the ≤ 10 yrs age group with the most common clinical presentation being a previous diagnosis of tuberculosis or recurrent upper respiratory tract infections. The predominant findings on chest radiographs were deviation of the trachea to the affected side, volume loss in the ipsilateral lung field, and compensatory hyperinflation of the contralateral lung field. On the CECT chest, the main findings were interrupted pulmonary artery, hypoplastic lung fields with bronchiectasis, or ground glassing. Associated cardiovascular and pulmonary malformations were identified with notable cases: TOF, right-sided aortic arch and scimitar syndrome. Their typical imaging findings have been elucidated and discussed in detail.

Conclusions

Patients with recurrent respiratory infections or hemoptysis having hypoplastic lung field with hyperinflation of the contralateral lung on chest radiographs should be evaluated for pulmonary artery interruptions. Chest CECT allows evaluation of the bronchial tree and lung parenchyma at the same time which helps distinguish pulmonary interruption from conditions such as Swyer-James-Macleod syndrome, pulmonary hypoplasia, thromboembolism and arteritis. Cases with PIPA can also be associated with cardiovascular and pulmonary anomalies such as TOF, partial anomalous pulmonary venous connection (PAPVC), and VSD. The knowledge of these associations is essential as they can influence the mode of treatment and can help reduce the long-term morbidity and mortality associated with the condition.

## Introduction

Proximal interruption of pulmonary artery (PIPA) is identified by an aberrant termination of the pulmonary artery at the hilum, resulting in a variety of clinical manifestations. Interruption of the right or left or bilateral pulmonary arteries can be identified in symptomatic patients or can be discovered incidentally in asymptomatic individuals who undergo evaluation with echocardiography or chest computed tomography (CT) performed as a part of regular health checks or part of evaluation for other nonassociated conditions [1]. Patients with isolated proximal interruptions usually can be asymptomatic until late adulthood. Clinically they present with dyspnea, chest discomfort, hemoptysis, and recurring infections as common symptoms [2]. We present a spectrum of cases with isolated PIPA and cases associated with other cardiovascular and pulmonary abnormalities. Identification of these cases includes a few typical imaging findings on chest radiographs and chest CT studies. The associated cardiovascular anomalies are best appreciated with a contrast-enhanced CT (CECT) of the chest. We have discussed and demonstrated the typical radiological imaging findings in detail.

## Materials and methods

Malla Reddy Medical College for Women Institutional Ethics Committee issued approval MRMCWIEC/AP/90/2022. Nineteen patients having an arrest of the pulmonary artery as a component of the findings in their chest CECT reports between 2019 to 2022 were taken as the study population (Table 1). Their case history, chest CT and chest radiographs were collected retrospectively. Chest radiographs were taken on two Allenger's 100 mA X-Ray machines and Fujifilm DR Cassettes. CECT and plain computed tomographs were done using General Electric (GE) 128 Slice Revolution EVO CT and Phillips 64 slice Brilliance CT machines. 

**Table 1:** Inclusion and exclusion criteria CECT: contrast-enhanced computed tomography

Inclusion Criteria	Exclusion Criteria
Cases with proximal arrest of pulmonary artery as a component of their Chest CECT reports between 2019 to 2022.	Cases which are already operated as a part of surgical management.

**Figure 1:**
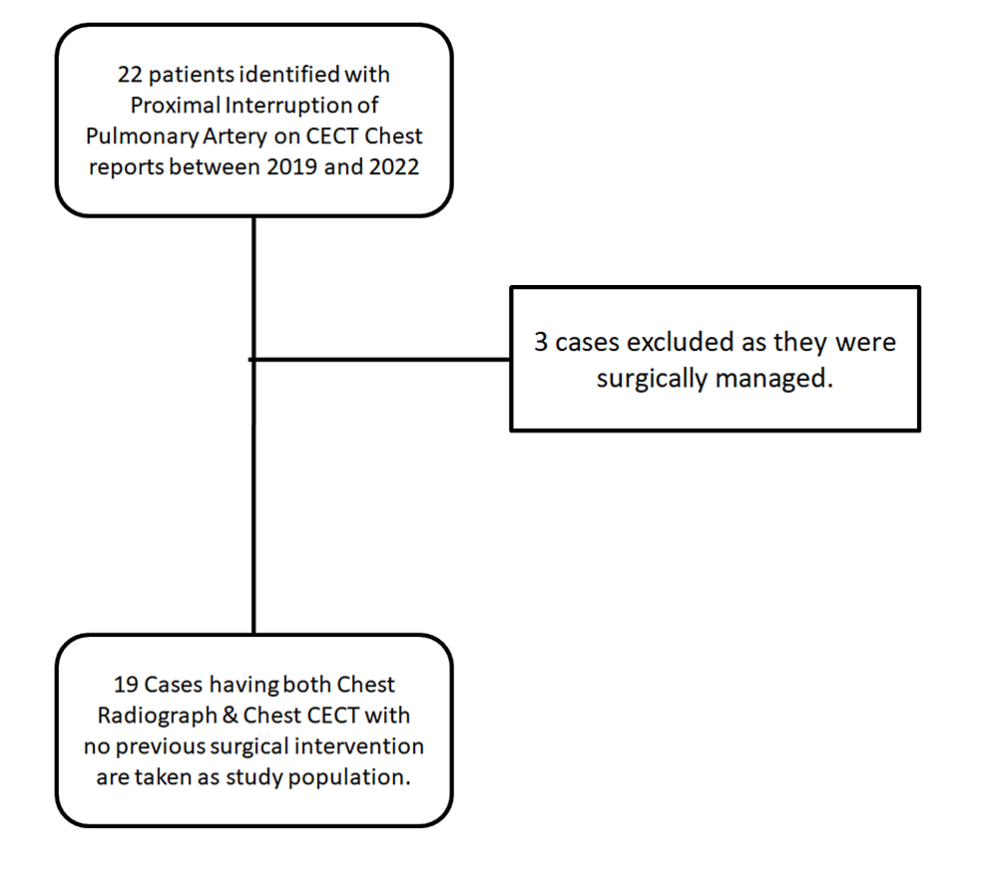
Sequence of steps involved in selection of cases. CECT: contrast-enhanced computed tomography

A total of 22 cases were identified with proximal arrest of pulmonary artery as a component of their chest CECT reports between 2019 and 2022. Three cases were excluded of as they were previously operated with transcatheter embolization for emergency management of massive hemoptysis and surgical correction as prosthetic anastomosis. Nineteen cases were taken as the study population (Figure 1).

Data were analyzed using Microsoft Excel 2019 (Microsoft, Redmond, WA, USA). Statistics regarding the demographic data, presenting complaints, arrested pulmonary artery, and associated cardiovascular were calculated.

## Results

Of the 19 cases, most of them belonged to the ≤10 years age group which is about 31.5% (n = 6) followed by 21% (n = 4) of them who belong to the 20-30 years age group (Table 2). 57.9% (n = 11 )were females and 42.1% (n = 8) were males. The most common clinical presentation was the previous history of tuberculosis which constituted 42% (n = 8) of the cases followed by recurrent upper respiratory tract infections in about 31.6% of the cases (n = 6) and breathing difficulty in 26.3% of cases (n = 5) (Table 3).

**Table 2:** Age distribution of the cases selected for the study.

Min (years)	Max (years)	Count	%
Less than 10	10	6	31.58
10	20	3	15.79
20	30	4	21.05
30	40	2	10.53
40	50	1	5.26
50	60	2	10.53
60	70	1	5.26
70	and above	0	0
TOTAL		19	

**Table 3:** Distribution of the presenting clinical symptoms in the selected cases.

Complaint	Count	Percentage
Breathing Difficulty	5	26.30%
Repeated Upper Respiratory Tract Infections (URTI)	6	31.60%
Diagnosed Tuberculosis	8	42.10%
Total	19	100%

The region of our hospital, being part of an endemic area for tuberculosis, makes tuberculosis the most common clinical finding in our study. Recurrent upper respiratory tract infections (URTI) were the second most common reason accounting for 31.6% of the cases which is almost similar to the findings of Reading et al. [1] who found the incidence of recurrent infections in about 37% of the patients. 

Chest radiograph findings

The most common findings on chest radiographs were deviation of the trachea to the affected side, volume loss in the ipsilateral lung field, and compensatory hyperinflation of the contralateral lung field with few of them showing ground-glass opacities. The differentials for tracheal deviation to the ipsilateral side include atelectasis and pleural fibrosis. Unilateral lung hyperinflation is also seen in asthma, chronic obstructive pulmonary disease (COPD), bronchiectasis and bronchiolitis. These differentials have to be ruled out while considering pulmonary agenesis as a possibility as a result of PIPA.

CECT chest findings

Pulmonary Artery Findings

Hypoplasia/atresia of the left pulmonary artery was found in 52.6% (n = 10) of the cases followed by hypoplasia/atresia of the right pulmonary artery in 31.6% (n = 6) cases. In two cases, bilateral pulmonary arteries were absent with one case having truncus arteriosus. An absent main pulmonary artery was noted in one case (Table 4). 

**Table 4:** Distribution of the involvement of pulmonary arteries in the present cases.

Pulmonary Artery Involved	Count	Percentage
Right	6	31.60%
Left	10	52.60%
Both	2	10.50%
Main	1	5.30%
Total	19	100%

Pulmonary Parenchymal Findings

In most of the cases, volume loss involving the ipsilateral lung was seen. Hypoplasia of bilateral pulmonary arteries and the main pulmonary artery was associated with normal bilateral lung fields.

In 68.4% of the cases (n = 13), cystic bronchiectasis was present. The other less common findings were honeycombing and centrilobular emphysema. 

Alveolar Opacification Pattern

Major alveolar opacification patterns identified were patchy nonspecific ground glass opacities and diffuse ground glassing. Diffuse pulmonary plethora was present in cases with absent bilateral pulmonary arteries and in patients with the absent main pulmonary artery.

Cardiovascular Findings

**Figure 2:**
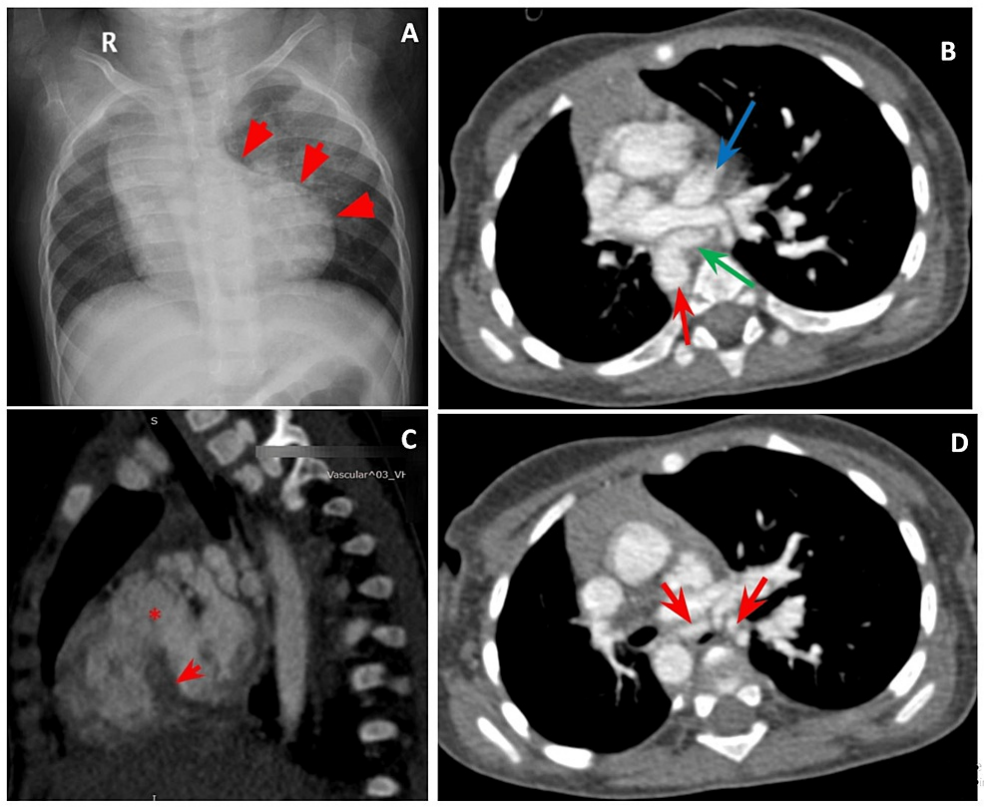
Proximal interruption of main pulmonary artery and bilateral pulmonary arteries with right sided aorta, MAPCAs and tetralogy of Fallot anomaly. A one-and-a-half-year-old female with complaints of irregular breathing, flaring of nostrils and episodes of sneezing. On further evaluation with Chest X-Ray to rule out the causes for difficulty of breathing tetralogy of Fallot was suspected and further evaluation with Chest CECT was done to identify associated cardiovascular and pulmonary anomalies. On Chest CECT, the main pulmonary artery and bilateral pulmonary arteries were not visualised, aorta was noted to course on the right side, few MAPCAS were identified, thick interventricular septum and a ventricular septal defect were identified. 2A: Chest Radiograph of the baby showing a rounded elevated cardiac apex suggestive of right ventricular hypertrophy with a boot shaped configuration of the heart (red arrows) - Features suggestive of tetralogy of Fallot anomaly. 2B: CECT scan of the thorax shows non-visualization of the main pulmonary artery, right main pulmonary artery, and left pulmonary artery. A left atrial diverticulum (blue arrow), right-sided aorta (red arrow) and a MAPCA (green arrow). 2C: CECT scan of the thorax shows a thick interventricular septum (arrow) with ventricular septal defect (asterisk). 2D: CECT scan of the thorax shows few arterial channels arising from the lateral aspect of descending aorta supplying pulmonary parenchyma - Features representing MAPCAs MAPCAs: Major Aorto-Pulmonary Collaterals, CECT: Contrast Enhanced Computed Tomography

Major aortopulmonary collateral arteries (MAPCAs) supplying both lungs were seen in one case with absent or atretic bilateral pulmonary arteries. In the other case, it is observed that MAPCAs were seen supplying the right lung with collateral from the left brachiocephalic trunk supplying the left lung. Both cases were identified with ventricular septal defects (VSD), right ventricular (RV) hypertrophy, and overriding aorta, together constituting tetralogy of Fallot (TOF) anomaly. The second case also had an associated right-sided aortic arch (Figure 2). One case noted an isolated finding of MAPCAs supplying the right lung in the absence of the right pulmonary artery.

**Figure 3:**
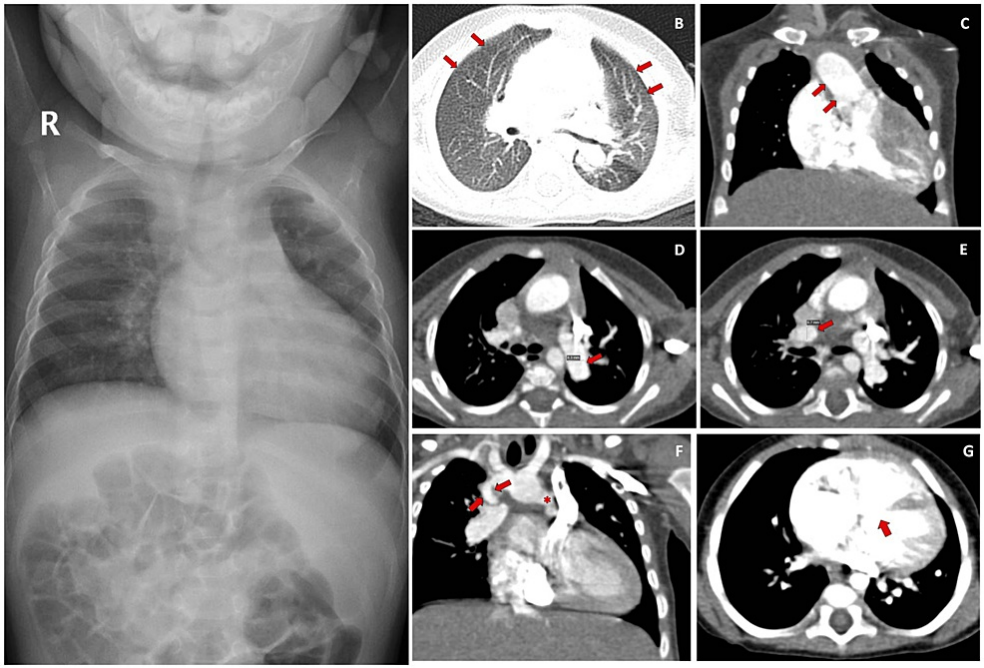
Proximal arrest of bilateral pulmonary arteries having truncus arteriosus anomaly with anomalous arterial channels from pulmonary artery and aorta supplying bilateral lungs. An 8-month-old male child was being evaluated for difficulty of breathing. As a routine part of protocol, Chest X-Ray was ordered which showed a widened mediastinum. A Chest CECT was ordered for further evaluation. The findings of Chest CECT as described below. 3A: Chest X-Ray showing widened mediastinum. 3B: Chest CT scan showing bilateral pulmonary plethora (red arrows). 3C: Chest CECT showing a common trunk (red arrows) connected with the aorta and pulmonary vasculature. 3D: Chest CECT demonstrating a hypoplastic left pulmonary artery (red arrow), 3E: Chest CECT demonstrating a hypoplastic right pulmonary artery (red arrow), 3F: Chest CECT demonstrating a right pulmonary artery arising from the brachiocephalic trunk (arising inferiorly) (red arrows) and small arterial channel (forming the left PA) seen arising from the aorta and supplying the left lung (asterisk). 3G: Chest CECT demonstrating a sub-aortic ventricular septal defect. PA: Pulmonary Artery, CECT: Contrast Enhanced Computed Tomography

Truncus arteriosus anomaly was found in one case, which was associated with an atretric main pulmonary artery with the right pulmonary artery arising from the brachiocephalic trunk and the left pulmonary artery was noted to arise from a vascular channel from the arch of the aorta. VSD was also noted in this case (Figure 3).

In three cases showing atresia of the right pulmonary artery, associated with hypoplasia of the right lung and cystic bronchiectasis, the right lung was supplied by extrapulmonary arteries from the aorta. One of the three cases with extrapulmonary arteries also was noted with a patent ductus arteriosus.

**Figure 4:**
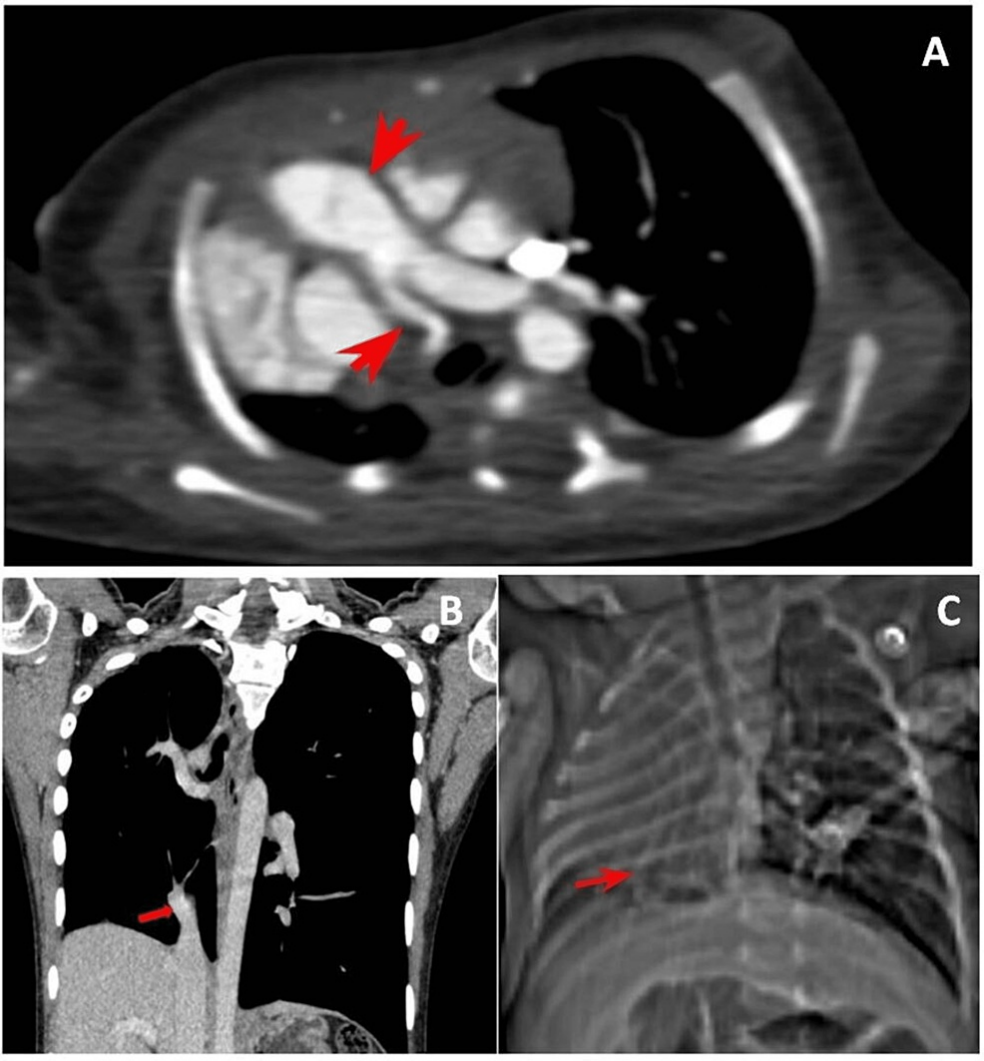
Hypoplastic right pulmonary artery with PAPVR forming the scimitar sign A 15-year-old female patient presented with complaints of repeated URTI. On further evaluation, hypoplastic right lung field was noted on Chest Radiograph. On Chest CECT evaluation, hypoplastic right pulmonary artery was noted with an anomalous vein draining into the IVC forming the scimitar sign. 4A: Chest CECT of the patient demonstrating a hypoplastic right pulmonary artery (red arrows). 4B: Chest CECT of the patient demonstrating an anomalous vein draining into the IVC (red arrow) suggestive of PAPVR - scimitar sign. 4C: Chest X-Ray of a 20-day neonate showing the scimitar sign (red arrow). PAPVR: Partial Anomalous Pulmonary Venous Return, URTI: Recurrent Upper Respiratory Tract Infection, IVC: Inferior Vena Cava

Two cases who had atresia of the right pulmonary artery with hypoplasia of the right lung and cystic bronchiectasis, also had a partial anomalous pulmonary venous return/connection (PAPVC) in the form of anomalous pulmonary veins on the right draining into inferior vena cava (Figure 4). This is presented as the scimitar sign(Figure 4C).

**Figure 5:**
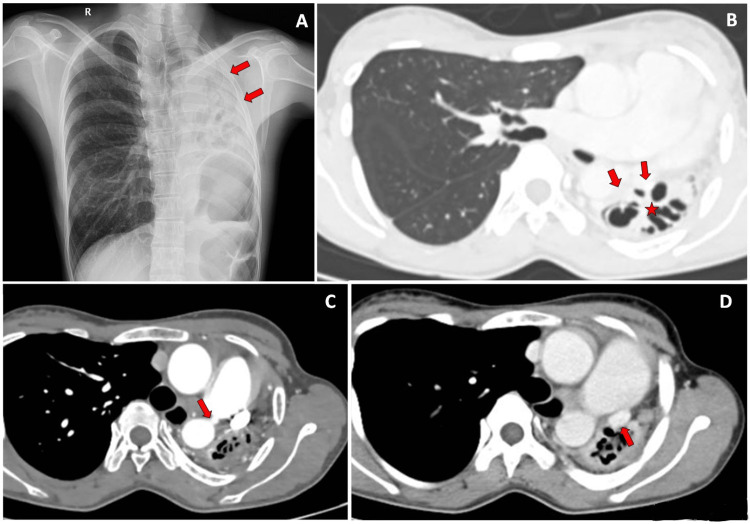
Proximal arrest of left pulmonary artery with patent ductus arteriosus anomaly. A 20-year-old female presented with complaints of recurrent upper respiratory tract infections. On Chest Radiography, hypoplastic left upper lobe was noted. On CECT Chest examination, hypoplastic left upper lobe with cystic bronchiectasis was noted. The ductus arteriosus was noted to be patent along with a persistent left superior vena cava. 5A: Chest X-Ray shows hypoplastic left lung (red arrows). 5B: Chest CECT of the patient demonstrates hypoplastic left lung (red arrow) with cystic bronchiectasis (red star). 5C: Chest CECT of the patient demonstrates patent ductus arteriosus (red arrow). 5D: Chest CECT of the patient demonstrates persistent left superior vena cava (red arrow). CECT: Contrast Enhanced Computed Tomography

Patent ductus arteriosus was identified in two cases with one case showing a persistent left superior vena cava draining to the coronary sinus (Figure 5). Distribution of cardiovascular manifestations in the 19 cases presenting with arrest of pulmonary artery is shown in Table 5.

**Table 5:** Distribution of cardiovascular manifestations in the 19 cases presenting with arrest of pulmonary artery. Total cases with cardiovascular anomalies (n = 10) PAPVC: Partial anomalous pulmonary venous connection

Cardiovascular Anomaly	Count	Percentage
Major pulmonary collateral arteries (MAPCAS)	3	30%
Tetralogy of Fallot with MAPCAS	2	20%
Truncus Arteriosus	1	10%
Extrapulmonary Arteries	3	30%
PAPVC with Scimitar	1	10%

## Discussion

Proximal interruption of the pulmonary artery is a rare developmental anomaly with an incidence of 1 in 200,000 individuals [1]. It is characterized by short-segment atresia of the proximal left, right, or bilateral pulmonary arteries. Distal segments of the arteries in the hila and the lung are usually present. “Interruption” is a term that has also been used by several authors for this condition [2]. Earlier, the term "absence of pulmonary artery" was widely used for this condition. But, the recent consensus in scientific literature states that as the intrapulmonary vascular network is still intact, the term “interruption of the pulmonary artery” is preferred over “absence of pulmonary artery” [3]. 

Though the pulmonary artery is interrupted, the intraparenchymal arterial tree typically develops with blind ends proximally [4]. This arterial tree receives collateral blood supply from systemic vessels including the internal mammary artery, innominate artery, and bronchial artery [5,6].

Lungs develop as ventral buds from the embryonic esophagus. They receive blood supply from the visceral plexus that surrounds the esophagus. The plexus joins the proximal limb of the sixth aortic arch. The proximal portions of bilateral sixth aortic arches form the main pulmonary artery, left, and right pulmonary arteries. The distal portion of the sixth arch persists as ductus arteriosus on the left side while it atrophies on the right side [7].

On plain radiographs, hypoplasia of the lung can present as elevation of the diaphragm with an ipsilateral mediastinal shift indicating volume loss in the hemithorax [3]. Compensatory hyperinflation of the opposite lung with herniation into the smaller hemithorax can also be noted. In patients with collateral supply from dilated intercostal arteries, fine linear opacities can be noted in the peripheries of the lung [6]. Other findings can be the absence of hilar pulmonary artery shadow with the prominence of contralateral hilum, unilateral pulmonary edema, notching of ribs, and cardiovascular anomalies. The absence of hilar shadow can be noted in all the cases in this series.

On CT scans, the mediastinal portion of the affected pulmonary artery may be completely absent or may terminate within 1 cm of its origin [3]. Anastomosis of the transpleural collateral vessels with the peripheral branches of the pulmonary artery can be seen as serrated thickening of the pleura and subpleural parenchyma on CT scans [3]. A small hemithorax can be noted with compensatory hypertrophy of the opposite lung and resultant herniation of that lung into the ipsilateral hemithorax. 

Harkel et al. found that congenital unilateral absence of a pulmonary artery (UAPA) was also found to be associated with cardiovascular anomalies like tetralogy of Fallot, septal defect, subvalvular aortic stenosis, transposition of the great arteries, Taussig-Bing malformation and coarctation, pulmonary stenosis and scimitar syndrome [8]. 

MAPCAs are arteries that arise from the aorta and other systemic arteries to supply the lungs in cases with anomalous pulmonary artery development. They are commonly associated with TOF anomaly constituting pulmonary stenosis, a ventricular septal defect, right ventricular hypertrophy, and an overriding aorta.

Two of our patients had the proximal arrest of bilateral pulmonary arteries associated with the TOF anomaly. A few MAPCAs were seen arising from the right lateral aspect of descending aorta supplying the right pulmonary parenchyma.

We also present a rare association of right pulmonary artery interruption with persistent left superior vena cava and anomalous vein draining into inferior vena cava forming the scimitar syndrome with the typical scimitar sign.

Most of the patients remain asymptomatic till the late stage. However, the clinical symptoms may include recurrent pulmonary infections, decreased exercise tolerance, and mild dyspnoea during exertion [9]. The infections can rarely lead to complications such as necrotizing bronchopneumonia further resulting in neonatal pneumonectomy [10]. The cause of recurrent infections is attributed to hypocapnia leading to bronchoconstriction and decreased mucociliary clearance [11].

Limitations of the study

Our study was conducted in a tertiary care hospital. Since it is a referral center and PIPA can also be asymptomatic, the incidence of PIPA during the study period cannot truly represent the overall incidence of PIPA cases in this region. Since this was a cross-sectional study that collected data at a single point in time in the life of the patient, the progression of symptoms, lifetime morbidity, and mortality could not be evaluated. Further follow-up of these cases for long-term outcomes can add more knowledge to the available scientific literature regarding the morbidity and mortality burden associated with these anomalies.

## Conclusions

Patients with recurrent respiratory infections or hemoptysis presenting with hypoplastic lung and hyperinflation of the contralateral lung on chest radiographs should be further evaluated for pulmonary artery interruptions.

Contrast CT allows evaluation of the bronchial tree and lung parenchyma simultaneously which helps distinguish pulmonary interruption from Swyer-James-Macleod syndrome, pulmonary hypoplasia, thromboembolism, and arteritis.

These cases can also be associated with cardiovascular and pulmonary anomalies such as TOF, PAPVC, VSD, etcetera. The knowledge of these associations is important in the present and future management of these cases.

As these associations can be identified with chest CECT, unless embolization is required for control of hemoptysis, contrast CT eliminates the requirement for angiography making it the diagnostic modality of choice and hence, must be included in the evaluation protocol of such cases.
